# Effects of standing Baduanjin exercise on cardiac function and quality of life in patients with chronic heart failure: a systematic review and meta-analysis

**DOI:** 10.3389/fcvm.2026.1732696

**Published:** 2026-02-04

**Authors:** Yinli Jiao, Xiaoqi Gong, Yao Xiao, Yixuan Li, Ruogu Yang, Lipeng Shi, Lanlan Zhang

**Affiliations:** 1 Bishan Hospital, Chongqing University of Chinese Medicine, Chongqing, China; 2 Chongqing University of Chinese Medicine, Chongqing, China; 3 The First Affiliated Hospital of Chongqing University of Chinese Medicine, Chongqing, China

**Keywords:** cardiac function, chronic heart failure, meta-analysis, quality of life, standing Baduanjin exercise, systematic review

## Abstract

**Background:**

Chronic heart failure (CHF) is characterized by progressive cardiac dysfunction and impaired quality of life (QoL), which contribute to poor prognosis and frequent hospital readmissions. Standing Baduanjin exercise (SBE), a standing-form traditional Chinese mind–body exercise that integrates gentle movements, breathing control, and mental focus, has been increasingly applied in cardiac rehabilitation in China. However, although a growing number of randomized controlled trials (RCTs) have investigated SBE, a comprehensive synthesis of high-quality evidence regarding its effects on cardiac function and QoL in patients with CHF remains limited.

**Purpose:**

To evaluate the effects of SBE on cardiac function and QoL in patients with CHF.

**Methods:**

Following PRISMA and Cochrane guidelines, eight databases were searched for RCTs published up to October 2025. Study quality was assessed using the Cochrane Risk of Bias tool and modified Jadad scale. Meta-analysis was performed using Stata version 18.0, with subgroup analyses conducted based on intervention duration. Sensitivity analysis was performed by sequentially excluding individual studies, and publication bias was evaluated using Egger's test.

**Results:**

Fifty RCTs involving 3,964 participants were included. Compared with conventional pharmacotherapy (CPT) alone, SBE significantly improved left ventricular ejection fraction (LVEF: SMD = 0.98, 95% CI: 0.80–1.15, *P* = 0.000), 6 min walking distance (6-MWD: SMD = 1.20, 95% CI: 0.97–1.43, *P* = 0.000), and clinical efficacy (RR = 3.82, 95% CI: 2.83–5.17, *P* = 0.000), while reducing left ventricular end-diastolic diameter (LVEDD: SMD = −1.03, 95% CI: −1.29 to −0.76, *P* = 0.000), left ventricular end-systolic diameter (LVESD: SMD = −0.74, 95% CI: −0.96 to −0.52, *P* = 0.000), B-type natriuretic peptide (BNP: SMD = −1.36, 95% CI: −1.77 to −0.96, *P* = 0.000), and N-terminal pro-B-type natriuretic peptide (NT-pro BNP: SMD = −1.11, 95% CI: −1.41 to −0.82, *P* = 0.000). Furthermore, SBE significantly decreased total and subdomain scores of the Minnesota Living with Heart Failure Questionnaire (MLHFQ), including the total score (SMD = −1.17, 95% CI: −1.38 to −0.96, *P* = 0.000), psychological (SMD = −1.89, 95% CI: −2.43 to −1.34, *P* = 0.000), emotional (SMD = −1.86, 95% CI: −2.08 to −1.63, *P* = 0.000), and other domains (SMD = −2.04, 95% CI: −2.73 to −1.35, *P* = 0.000). Subgroup analyses indicated that a 12-week intervention achieved the greatest improvement in cardiac function, while interventions longer than 12 weeks produced the most pronounced enhancement in QoL.

**Conclusion:**

Current evidence suggests that SBE is an effective, safe, and practical adjunctive intervention for CHF, capable of improving both cardiac function and QoL. Further high-quality multicenter RCTs are warranted to confirm these findings and explore long-term outcomes.

**Systematic Review Registration:**

https://www.crd.york.ac.uk/PROSPERO/view/CRD420251175888, identifier CRD420251175888.

## 1 Introduction

Chronic heart failure (CHF) is a major global public health concern characterized by high morbidity, mortality, and readmission rates (1). According to the Global Burden of Cardiovascular Diseases (GBD) 2023 report, cardiovascular diseases account for approximately 19.2 million deaths worldwide and over 437 million disability-adjusted life years (DALYs), with heart failure being one of the leading contributors to both mortality and disability (2). The global prevalence of heart failure is estimated at 1%–2%, exceeding 10% among individuals aged 70 years or older, and the average age of onset continues to decline (3). Although pharmacological and device-based therapies—such as *β*-blockers, angiotensin-converting enzyme inhibitors (ACEIs), angiotensin receptor-neprilysin inhibitors (ARNIs), and implantable cardiac devices—have significantly improved survival outcomes, many patients still experience reduced exercise tolerance, fatigue, depression, and impaired quality of life (QoL) (4–6). The chronic and progressive nature of CHF necessitates long-term management strategies that extend beyond medication, emphasizing safe, cost-effective, and sustainable integrative interventions to further enhance cardiac function and overall health.

Cardiac dysfunction is the central pathophysiological feature of CHF, leading to reduced cardiac output, inadequate tissue perfusion, and limited exercise capacity. These physiological impairments not only manifest as fatigue and dyspnea but also contribute to psychosocial issues such as anxiety, depression, and social withdrawal, which substantially impair patients' QoL (7). Clinically, QoL in CHF is commonly assessed using the Kansas City Cardiomyopathy Questionnaire (KCCQ) and the Minnesota Living with Heart Failure Questionnaire (MLHFQ)—the former being more sensitive to short-term interventions, while the latter reflects long-term quality of life and prognosis (8). Studies have shown that approximately 60% of CHF patients suffer from markedly reduced QoL (KCCQ < 60 or MLHFQ > 45) (9, 10). QoL deterioration is closely associated with cardiac dysfunction, as evidenced by the negative correlation between left ventricular ejection fraction (LVEF) and QoL scores, and the positive correlation between N-terminal pro-B-type natriuretic peptide (NT-pro BNP) levels and disease severity (11). Importantly, QoL serves not only as a measure of disease burden but also as an independent prognostic indicator (12). Previous studies have demonstrated that each 5-point increase in KCCQ score is associated with a significant reduction in mortality and rehospitalization risk (13). Therefore, improving both cardiac function and quality of life has become the dual focus of CHF management.

Baduanjin, a traditional Chinese mind-body exercise with a history of over 800 years, is one of the rehabilitation practices officially recommended by the General Administration of Sport of China (14). Baduanjin primarily exists in two forms: the standing Baduanjin, which emphasizes dynamic full-body movements and cardiopulmonary activation; and the sitting Baduanjin, which focuses on gentle upper-limb exercises combined with breath regulation (15). As a typical low- to moderate-intensity mind-body intervention, Baduanjin exerts its rehabilitative effects not only through muscular activity and aerobic conditioning but also via comprehensive modulation of the nervous, autonomic, immune, and circulatory systems (16). Rhythmic breathing and progressive stretching during Baduanjin practice enhance vagal tone and suppress sympathetic over activity, thereby improving heart rate variability (HRV) and cardiac autonomic regulation (17). Long-term training has been shown to attenuate systemic inflammation and oxidative stress, as evidenced by reductions in C-reactive protein (CRP), interleukin-6 (IL-6), and tumor necrosis factor-α (TNF-α), along with increased activity of antioxidant enzymes such as superoxide dismutase (SOD) and glutathione peroxidase (GSH-Px) (18). These physiological adaptations help mitigate myocardial injury and fibrosis progression. Moreover, the deep breathing and postural extension components of Baduanjin can stimulate endothelial nitric oxide (NO) production, improve endothelium-dependent vasodilation and microcirculatory perfusion, and optimize cardiac preload, afterload, and oxygen supply (19). Collectively, these mechanisms synergistically enhance myocardial pump function and cardiopulmonary endurance, providing a physiological foundation for long-term rehabilitation in patients with CHF. However, most existing studies are limited by small sample sizes, methodological heterogeneity, and inadequate differentiation between different forms of Baduanjin practice. Therefore, this study aimed to conduct a systematic review and meta-analysis to quantitatively evaluate the effects of standing Baduanjin exercise on cardiac function and quality of life in patients with CHF.

## 2 Methods

This systematic review and meta-analysis aimed to evaluate the effects of standing Baduanjin exercise (SBE) combined with conventional pharmacological therapy (CPT) on cardiac function and QoL in patients with CHF. The study was conducted in strict accordance with the Cochrane Collaboration guidelines and the Preferred Reporting Items for Systematic Reviews and Meta-Analyses (PRISMA) statement (20) (Supplementary Material S1). The study protocol was prospectively registered with PROSPERO (Registration No.: CRD420251175888).

### 2.1 Search strategy

A comprehensive literature search was conducted across eight electronic databases—PubMed, Embase, Cochrane Library, Web of Science, Wanfang Data, Chinese Biomedical Literature Database (CBM), VIP Database, and China National Knowledge Infrastructure (CNKI)—to identify randomized controlled trials (RCTs) published from database inception to October 2025. Both Medical Subject Headings (MeSH) terms and free-text keywords were employed, with no restrictions on language. The core search terms included: “Baduanjin,” “Eight-Section Brocade,” “standing Baduanjin,” “chronic heart failure,” “heart failure,” “cardiac function,” and “quality of life”. Boolean operators (AND, OR, NOT) were applied to refine the search.

To ensure comprehensiveness, manual searches were also performed on the reference lists of all included articles to identify additional eligible studies. Two reviewers (YJ and YX) independently conducted the search and screening processes, and any discrepancies were resolved through discussion or consultation with a third reviewer (LS). Detailed search strategies for each database are provided in Supplementary Material S2.

### 2.2 Inclusion and exclusion criteria

The inclusion criteria were as follows:

Study design: randomized controlled trials (RCTs).
Participants: adults (≥18 years) diagnosed with CHF according to established diagnostic criteria, with New York Heart Association (NYHA) functional class II-III.
Interventions: the experimental group received SBE in combination with CPT, while the control group received CPT alone.
Primary outcomes: cardiac function indicators, including left ventricular ejection fraction (LVEF), left ventricular end-diastolic diameter (LVEDD), left ventricular end-systolic diameter (LVESD), B-type natriuretic peptide (BNP), N-terminal pro-B-type natriuretic peptide (NT-pro BNP), six-minute walking distance (6-MWD); and quality of life indicators, including total and domain scores (physical, psychological, and others) from the MLHFQ. Secondary outcome: clinical efficacy, defined as overall clinical improvement based on NYHA class and symptoms.
Data availability: studies providing sufficient quantitative data for extraction and analysis.

The exclusion criteria were as follows:

non-randomized, uncontrolled, or animal studies.
interventions involving sitting Baduanjin exercise or studies that did not clearly distinguish between standing and sitting forms.
incomplete follow-up data or missing outcome indicators.
duplicate publications or studies with overlapping data.

### 2.3 Data extraction and quality assessment

Two reviewers (YJ and XG) independently extracted data from all eligible studies, including the first author, year of publication, sample size, participant characteristics, intervention protocol (frequency, duration, and training period), control measures, and outcome indicators. Any discrepancies were resolved through discussion or adjudication by a third reviewer (LZ). The methodological quality of the included trials was assessed using the Cochrane Risk of Bias Tool (21), covering seven domains: random sequence generation, allocation concealment, blinding, completeness of outcome data, and selective reporting, among others. Additionally, the modified Jadad scale (total score = 7 points) was applied to evaluate study quality, with scores ≥4 indicating high-quality studies (22).

### 2.4 Statistical analysis

Meta-analyses were performed using Stata version 18.0. Dichotomous variables were expressed as risk ratios (RRs) with 95% confidence intervals (CIs), while continuous variables were reported as standardized mean differences (SMDs) with 95% CIs. Between-study heterogeneity was assessed using the *I*^2^ statistic. A random-effects model was applied when *I*^2^ > 50%, and a fixed-effects model was used otherwise. For continuous outcomes, a positive SMD indicates improvement for outcomes with higher-is-better values (e.g., LVEF and 6-MWD), whereas a negative SMD indicates improvement for outcomes with lower-is-better values (e.g., LVEDD, LVESD, BNP, NT-pro BNP, and MLHFQ).

Sensitivity analyses were conducted by sequentially omitting individual studies to evaluate the robustness of pooled results. Publication bias was assessed using Egger's test, and, when necessary, the trim-and-fill method was employed to adjust for potential bias. Additionally, subgroup analyses were performed according to intervention duration (<12 weeks, 12 weeks, and >12 weeks), based on commonly used exercise rehabilitation cycles, to explore the temporal effects of SBE on cardiac function and QoL outcomes.

## 3 Results

### 3.1 Literature search and study characteristics

**Figure 1:**
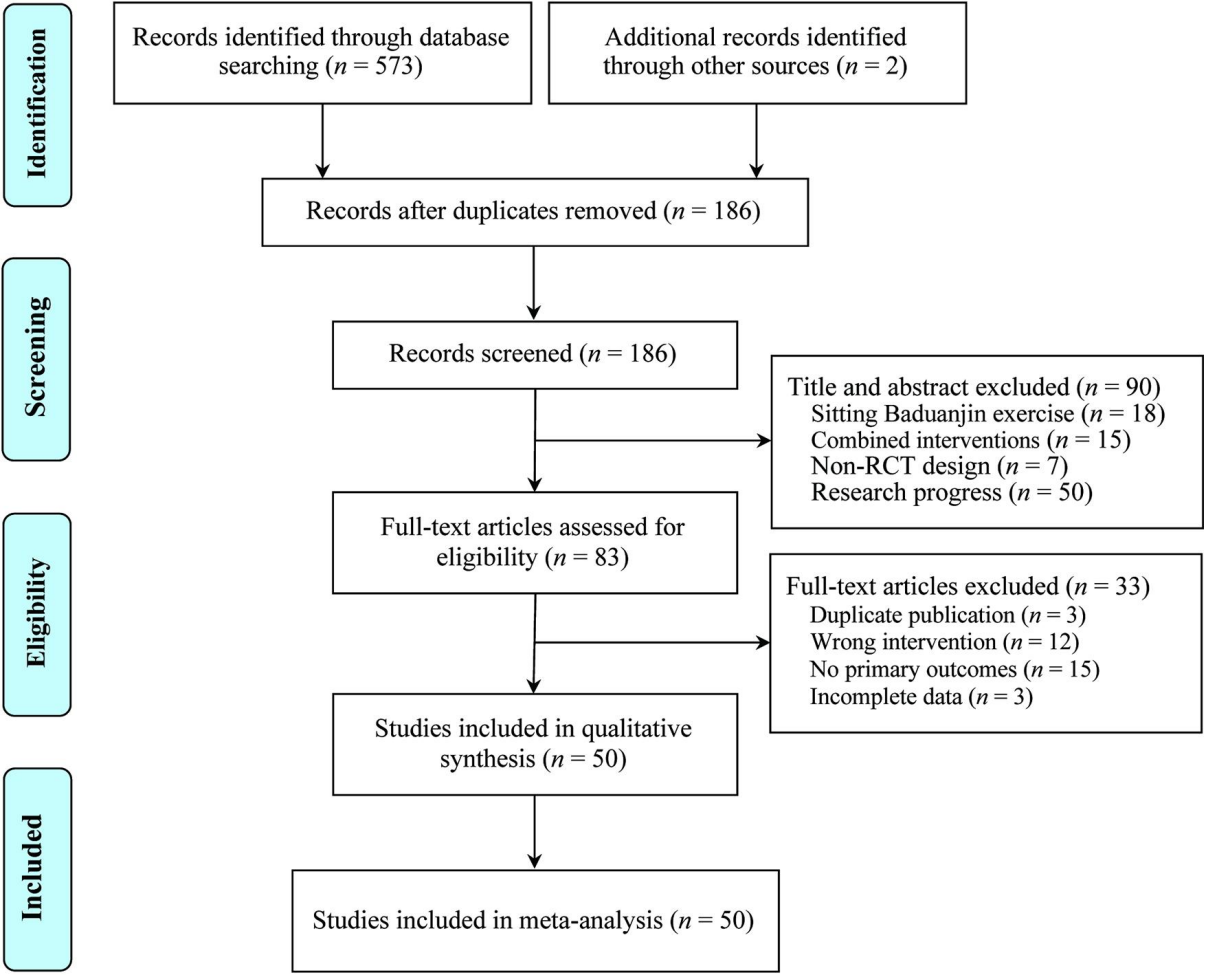
The PRISMA study ﬂowchart.

A total of 387 records were initially identified, of which 50 RCTs (23–72) met the inclusion criteria, encompassing 3,964 participants (2,219 males and 1,745 females) (Figure 1). All included studies were conducted in China and published between 2016 and 2025. Sample sizes ranged from 22 to 90 participants, with intervention durations varying from 2 to 48 weeks. All patients were classified as NYHA functional class II-III, and no adverse events related to SBE were reported. The control groups received CPT recommended by clinical guidelines, including ARNIs, ACEIs, angiotensin receptor blockers (ARBs), β-blockers, sodium-glucose cotransporter-2 inhibitors (SGLT2i), mineralocorticoid receptor antagonists (MRAs), and diuretics. The intervention groups performed SBEs in addition to these conventional therapies. No statistically significant differences were observed between the two groups at baseline. The main outcome measures included LVEF (39 studies), LVEDD (20 studies), LVESD (15 studies), 6-MWD (32 studies), BNP (11 studies), NT-pro BNP (18 studies), MLHFQ total score (24 studies), and its subdomains—physical (11 studies), psychological (10 studies), and other dimensions (8 studies)—as well as clinical efficacy (20 studies). The baseline characteristics of the included studies are summarized in Table 1.

**Table 1:** Included study characteristics.

Included studies	Sample size		Sex (M/F)		Mean age (years)		Course of disease (years)		Interventions		Treatment duration	Outcomes
	T	C	T	C	T	C	T	C	T	C		
Ai and Bian, (44)	28	30	13/15	12/18	67.71 ± 3.79	68.07 ± 4.80	9.21 ± 6.32	9.13 ± 3.44	SBE + CPT (ARNI, SGLT2i, MRA, *β*-blockers, diuretics)	CPT (ARNI, SGLT2i, MRA, *β*-blockers, diuretics)	24W	①②③⑤⑦⑪
Chen et al., (42)	71	71	43/28	41/30	61.69 ± 7.37	61.53 ± 5.28	5.54 ± 0.23	5.37 ± 0.17	SBE + CPT (ARNI, MRA, β-blockers, diuretics)	CPT (ARNI, MRA, β-blockers, diuretics)	12W	①②③④⑤
Chen, (43)	40	40	17/23	12/28	68.97 ± 2.31	68.86 ± 2.25	2.12 ± 0.35	2.09 ± 0.32	SBE + CPT + Routine nursing care	CPT + Routine nursing care	12W	④⑤⑧⑨⑩
Fang et al., (57)	22	22	9/13	13/9	72.14 ± 4.76	74.50 ± 4.02	-	-	SBE + CPT + Routine nursing care	CPT + Routine nursing care	12W	④⑤⑦
Gan, (41)	40	40	24/16	25/15	63.97 ± 4.94	64.27 ± 4.34	-	-	SBE + CPT	CPT	8W	①④⑤⑧⑨⑩
Gu and Zhang, (56)	34	34	20/14	19/15	67.00 ± 8.55	67.00 ± 8.50	-	-	SBE + CPT	CPT	24W	①②③④
Huang et al., (68)	24	24	11/13	12/12	57.45 ± 7.65	56.53 ± 7.46	10.36 ± 6.57	9.84 ± 5.26	SBE + CPT (digoxin, diuretics, nitrates)	CPT (digoxin, diuretics, nitrates)	3W	①②③⑤⑦⑪
Jiang and You, (55)	80	80	47/33	44/36	67.78 ± 4.10	67.23 ± 4.34	7.37 ± 3.16	7.55 ± 3.22	SBE + CPT	CPT	24W	①②③④⑥⑦⑧⑨
Jiao et al., (67)	40	40	18/22	19/21	66.6 ± 6.32	64.32 ± 5.23	3–8	3–8	SBE + CPT (ARNI, SGLT2i, MRA, β-blockers, diuretics, nitrates)	CPT (ARNI, SGLT2i, MRA, β-blockers, diuretics, nitrates)	24W	①④⑥⑦⑪
Kang et al., (64)	38	38	21/17	23/15	68.36 ± 5.03	69.25 ± 5.33	-	-	SBE + CPT (ARNI, SGLT2i, MRA, β-blockers, diuretics, nitrates, statins)	CPT (ARNI, SGLT2i, MRA, β-blockers, diuretics, nitrates, statins)	24W	①④⑥⑦
Li et al., (40)	50	50	29/21	32/18	65–72	59–72	6.24 ± 2.51	6.26 ± 2.56	SBE + CPT (ARNI, SGLT2i, MRA, β-blockers, diuretics)	CPT (ARNI, SGLT2i, MRA, β-blockers, diuretics)	24W	①②⑥
Li, (71)	30	30	18/12	20/10	69.24 ± 6.56	66.56 ± 5.31	3.56 ± 3.10	3.82 ± 3.67	SBE + SBE + CPT	CPT	8W	④⑦
Liu and Li, (39)	30	30	19/11	22/8	68.41 ± 2.39	66.74 ± 3.18	-	-	SBE + CPT (ARNI, MRA, β-blockers, diuretics, statins)	CPT (ARNI, MRA, β-blockers, diuretics, statins)	4W	①⑦⑪
Liu et al., (29)	30	30	20/10	19/11	64.30 ± 4.49	64.25 ± 4.63	7.79 ± 3.10	7.64 ± 3.24	SBE + CPT (ACEI, β-blockers, diuretics)	CPT (ACEI, β-blockers, diuretics)	12W	①②③④⑤⑪
Liu, (63)	48	48	26/22	25/23	63.29 ± 5.61	64.11 ± 5.63	4.39 ± 0.51	4.41 ± 0.53	SBE + CPT (ARNI, SGLT2i, MRA, β-blockers, diuretics)	CPT (ARNI, SGLT2i, MRA, β-blockers, diuretics)	12W	①②③⑦⑧⑨
Lv et al., (28)	30	30	16/14	16/14	49.81 ± 1.52	48.23 ± 2.56	-	-	SBE + CPT	CPT	20W	①②⑥⑪
Ma et al., (38)	60	60	31/29	32/28	64.17 ± 7.33	64.12 ± 7.36	5.55 ± 1.53	5.53 ± 1.56	SBE + CPT (ARNI, SGLT2i, MRA, β-blockers, diuretics)	CPT (ARNI, SGLT2i, MRA, β-blockers, diuretics)	12W	①②③④⑦⑪
Pan et al., (70)	42	42	22/20	23/19	68.28 ± 8.36	68.59 ± 8.14	3.62 ± 1.19	3.50 ± 1.23	SBE + CPT	CPT	8W	①④⑥
Peng, (37)	43	43	23/20	25/18	64.25 ± 3.78	64.78 ± 3.91	-	-	SBE + CPT (ARNI, SGLT2i, MRA, β-blockers, diuretics)	CPT (ARNI, SGLT2i, MRA, β-blockers, diuretics)	8W	①②③④⑪
Qi et al., (66)	50	50	34/16	37/13	62.8 ± 9.7	65.2 ± 7.8	7.1 ± 1.8	8.5 ± 2.1	SBE + CPT (digoxin, diuretics, nitrates)	CPT (digoxin, diuretics, nitrates)	12W	①②③⑧⑨⑩⑪
Sun et al., (47)	54	54	37/17	34/20	67.53 ± 2.41	68.11 ± 2.57	-	-	SBE + CPT	CPT	8W	①④⑤⑧⑨⑩
Sun, (69)	29	29	12/17	13/16	61.72 ± 7.50	60.34 ± 8.09	-	-	SBE + CPT	CPT	12W	①④⑥⑦⑪
Tu, (27)	23	23	14/9	13/10	74.06 ± 7.12	73.52 ± 6.78	-	-	SBE + CPT	CPT	2W	①④⑥⑦⑪
Wan et al., (65)	38	38	20/18	17/21	72.0 ± 3.2	73.4 ± 3.5	7.4 ± 4.1	7.2 ± 4.6	SBE + CPT (ARNI, SGLT2i, MRA, β-blockers, diuretics)	CPT (ARNI, SGLT2i, MRA, β-blockers, diuretics)	8W	④⑤⑦⑪
Wang and Pan, (62)	31	31	11/20	19/12	60.5 ± 8.0	62.1 ± 9.1	-	-	SBE + CPT + Routine nursing care	CPT + Routine nursing care	24W	④⑥⑦
Wang et al., (61)	40	40	26/14	25/15	66.7 ± 3.5	66.3 ± 3.7	-	-	SBE + CPT (ARNI, SGLT2i, MRA, β-blockers, diuretics)	CPT (ARNI, SGLT2i, MRA, β-blockers, diuretics)	3W	①④⑥
Wang et al., (46)	60	60	33/27	34/26	65.18 ± 9.92	62.55 ± 10.78	-	-	SBE + CPT	CPT	24W	①④⑥⑪
Wang, (54)	45	45	21/24	23/22	53.58 ± 7.12	52.36 ± 6.78	9.67 ± 5.87	10.35 ± 5.47	SBE + CPT	CPT	8W	①②③⑧⑨⑩
Wu et al., (26)	54	54	30/24	27/27	59.14 ± 10.72	60.28 ± 7.27	-	-	SBE + CPT (ARNI, SGLT2i, MRA, β-blockers, diuretics, nitrates, statins)	CPT (ARNI, SGLT2i, MRA, β-blockers, diuretics, nitrates, statins)	12W	①⑥⑪
Xiao et al., (36)	40	40	28/12	21/19	62.72 ± 5.78	63.64 ± 5.34	2.12 ± 0.88	2.31 ± 0.76	SBE + CPT (ACEI, β-blockers, diuretics)	CPT (ACEI, β-blockers, diuretics)	12W	①②③⑪
Xiong and Deng, (72)	33	30	20/13	18/12	70.3 ± 6.4	69.7 ± 7.2	-	-	SBE + CPT (ACEI, diuretics, nitrates, statins)	CPT (ACEI, diuretics, nitrates, statins)	12W	①④⑥⑧
Xu et al., (53)	45	44	24/21	25/19	70.12 ± 4.97	68.95 ± 5.67	-	-	SBE + CPT + Routine nursing care	CPT + Routine nursing care	4W	①④⑦
Yang et al., (52)	30	30	18/12	16/14	62.1 ± 9.1	61.5 ± 8.9	-	-	SBE + CPT (ACEI, β-blockers, diuretics)	CPT (ACEI, β-blockers, diuretics)	12W	④⑤⑥⑦
Yang et al., (32)	90	90	46/44	48/42	69.4 ± 8.1	68.6 ± 7.3	7.8 ± 2.3	7.7 ± 2.1	SBE + CPT	CPT	12W	①⑦⑧⑨⑩
Yang et al., (33)	43	43	23/20	26/17	58.76 ± 5.74	59.11 ± 6.05	3.01 ± 0.57	3.07 ± 0.64	SBE + CPT (ACEI, β-blockers, diuretics)	CPT (ACEI, β-blockers, diuretics)	2W	①⑪
Yang et al., (34)	30	30	17/13	16/14	58.85 ± 8.28	55.90 ± 0.75	5.62 ± 1.92	2.22 ± 1.15	SBE + CPT (ARNI, SGLT2i, MRA, β-blockers, diuretics, nitrates, statins)	CPT (ARNI, SGLT2i, MRA, β-blockers, diuretics, nitrates, statins)	12W	①④⑥
Yang et al., (35)	45	45	23/22	24/21	61.13 ± 7.93	62.18 ± 7.23	4.63 ± 0.98	4.25 ± 1.02	SBE + CPT + Routine nursing care	CPT + Routine nursing care	12W	①④⑤⑪
Ye et al., (60)	40	40	29/11	27/13	63.22 ± 18.72	65.12 ± 19.91	-	-	SBE + CPT + Routine nursing care	CPT + Routine nursing care	24W	⑦
Yu et al., (50)	30	30	13/17	16/14	54.93 ± 10.25	57.86 ± 10.82	8.82 ± 5.57	9.21 ± 5.51	SBE + CPT (ACEI, β-blockers, diuretics)	CPT (ACEI, β-blockers, diuretics)	2W	①④⑥⑦
Zhang et al., (24)	40	40	23/17	25/15	67.10 ± 9.80	65.40 ± 10.20	5.50 ± 2.90	5.20 ± 3.10	SBE + CPT (ARNI, SGLT2i, MRA, β-blockers, diuretics)	CPT (ARNI, SGLT2i, MRA, β-blockers, diuretics)	12W	①②③④⑤⑦
Zhao et al., (23)	40	40	22/18	21/19	58.32 ± 5.14	57.96 ± 5.37	-	-	SBE + CPT (ARNI, SGLT2i, MRA, β-blockers, diuretics)	CPT (ARNI, SGLT2i, MRA, β-blockers, diuretics)	48W	①②④⑦
Zhao et al., (24)	41	41	23/18	21/20	64.35 ± 7.76	63.18 ± 7.24	-	-	SBE + CPT	CPT	24W	①②⑥
Zheng, (31)	40	40	24/16	22/18	64.51 ± 4.50	64.25 ± 4.47	8.67 ± 2.28	8.33 ± 2.19	SBE + CPT (ACEI, β-blockers, diuretics)	CPT (ACEI, β-blockers, diuretics)	8W	①④⑦
Zhou et al., (59)	50	50	27/23	31/19	57.87 ± 4.61	58.41 ± 4.52	6.21 ± 1.24	5.67 ± 1.74	SBE + CPT	CPT	12W	①②③④⑧⑨⑩
Zhou et al., (49)	60	60	37/23	35/25	53.26 ± 5.14	53.84 ± 4.27	2.13 ± 0.48	2.09 ± 0.57	SBE + CPT	CPT	24W	①②③⑪
Zhu and Zhu, (30)	39	39	22/17	21/18	64.11 ± 7.76	64.62 ± 7.65	3.72 ± 1.52	3.46 ± 1.14	SBE + CPT (digoxin, ACEI, β-blockers, diuretics)	CPT (digoxin, ACEI, β-blockers, diuretics)	4W	①②④⑦⑪
Zhu et al., (45)	43	43	24/19	22/21	74.06 ± 3.81	73.96 ± 3.85	-	-	SBE + CPT + Routine nursing care	CPT + Routine nursing care	24W	④⑦⑧⑨⑩⑪

C, control group; T, treatment group; M, male; F, female; W, weeks; SBE: standing Baduanjin exercise; CPT, conventional pharmacological therapy; ACEI, angiotensin-converting enzyme inhibitor; ARNI, angiotensin receptor–neprilysin inhibitor; SGLT2i, sodium–glucose cotransporter-2 inhibitor; MRA, mineralocorticoid receptor antagonist; Outcomes: ① LVEF; ② LVEDD; ③ LVESD; ④ 6-MWD; ⑤ BNP; ⑥ NT-pro BNP; ⑦ MLHFQ total score; ⑧ Physical dimensions; ⑨ Psychological dimensions; ⑩ Other dimensions; ⑪ Clinical efficacy. -, not report.

### 3.2 Risk of bias assessment

**Figure 2:**
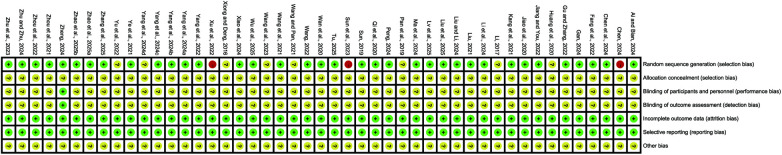
Bias risk assessment of included studies.

All 50 included RCTs reported their methods of randomization. Among them, 32 studies (24–31, 33–42, 44, 49, 54, 55, 57, 59, 60, 62–67, 69) used the random number table method, 3 studies (23, 45, 56) used lot-drawing, and 1 study (51) used sealed envelopes, all assessed as having low risk of bias. In contrast, 1 study (57) allocated participants based on order of admission, and 2 studies (43, 53) by treatment type, all considered to have high risk of bias. Eight studies (32, 46, 50, 61, 68, 70–72) did not specify the method of randomization and were rated as unclear. Only one study (31) explicitly reported the use of blinding and was assessed as low risk, whereas the remaining studies did not report details of blinding or allocation concealment and were thus rated as unclear. All studies reported complete outcome data, suggesting a low risk of attrition bias. None of the studies mentioned other potential sources of bias, and these were therefore classified as unclear. Detailed assessments of bias risk are presented in Figure 2 and Supplementary Material S3.

### 3.3 Outcomes

#### 3.3.1 LVEF

**Figure 3:**
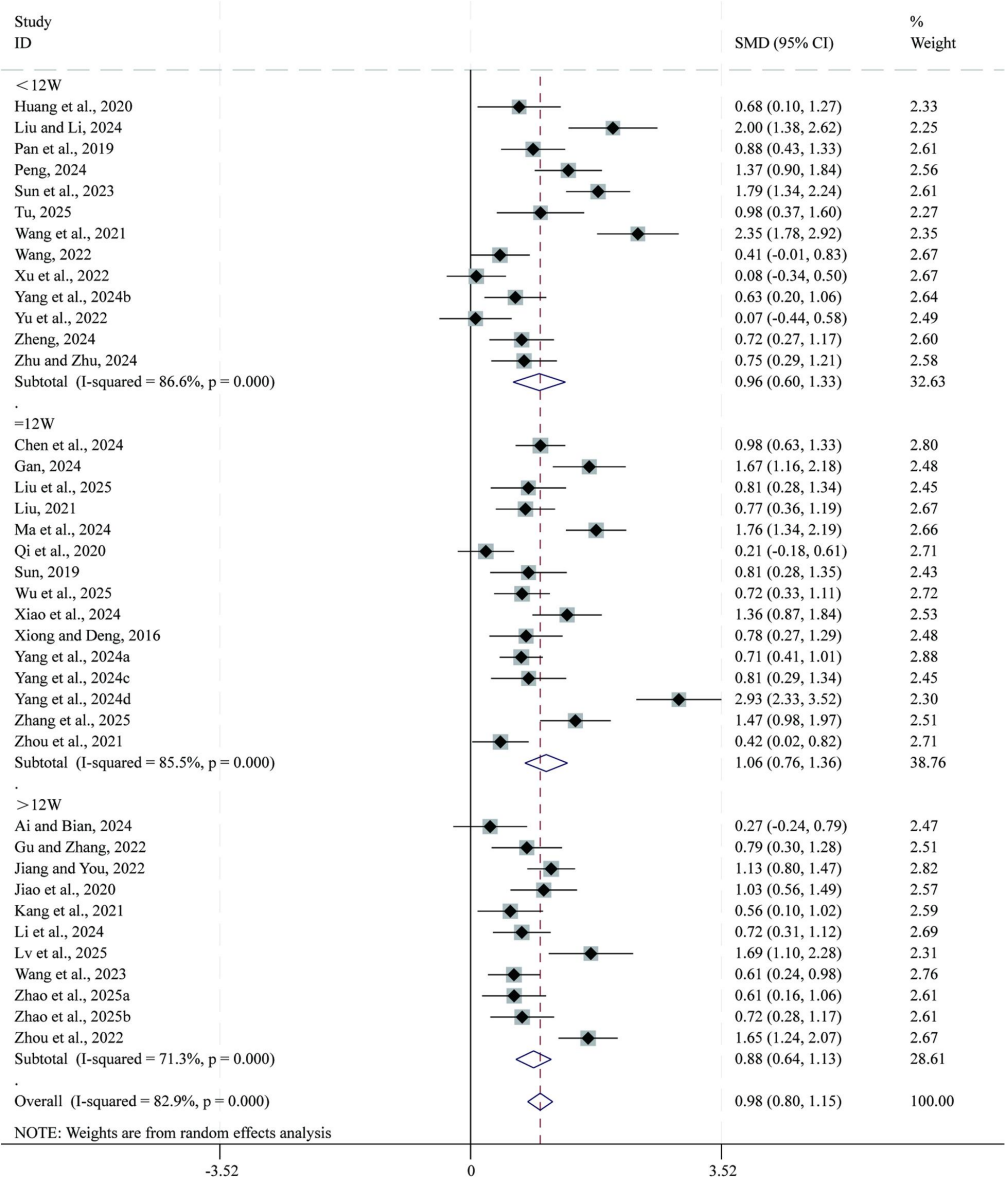
Forest plot for LVEF.

Total 39 studies (23–42, 44, 46, 47, 49, 50, 53–56, 59, 62–64, 66–70, 72) reported data on LVEF. Given the substantial heterogeneity across studies (*I²* = 82.9%, *P* = 0.000), a random-effects model was applied for the meta-analysis. Compared with CPT, SBE significantly improved LVEF (SMD = 0.98, 95% CI: 0.80–1.15, *P* = 0.000; Figure 3). Subgroup analysis based on intervention duration indicated that the greatest improvement in LVEF occurred in the 12-week intervention subgroup (SMD = 1.06, 95% CI: 0.76–1.36, *P* = 0.000), followed by the <12-week subgroup (SMD = 0.96, 95% CI: 0.60–1.33, *P* = 0.000), whereas the >12-week subgroup showed comparatively smaller improvements (SMD = 0.88, 95% CI: 0.64–1.13, *P* = 0.000).

#### 3.3.2 LVEDD

**Figure 4:**
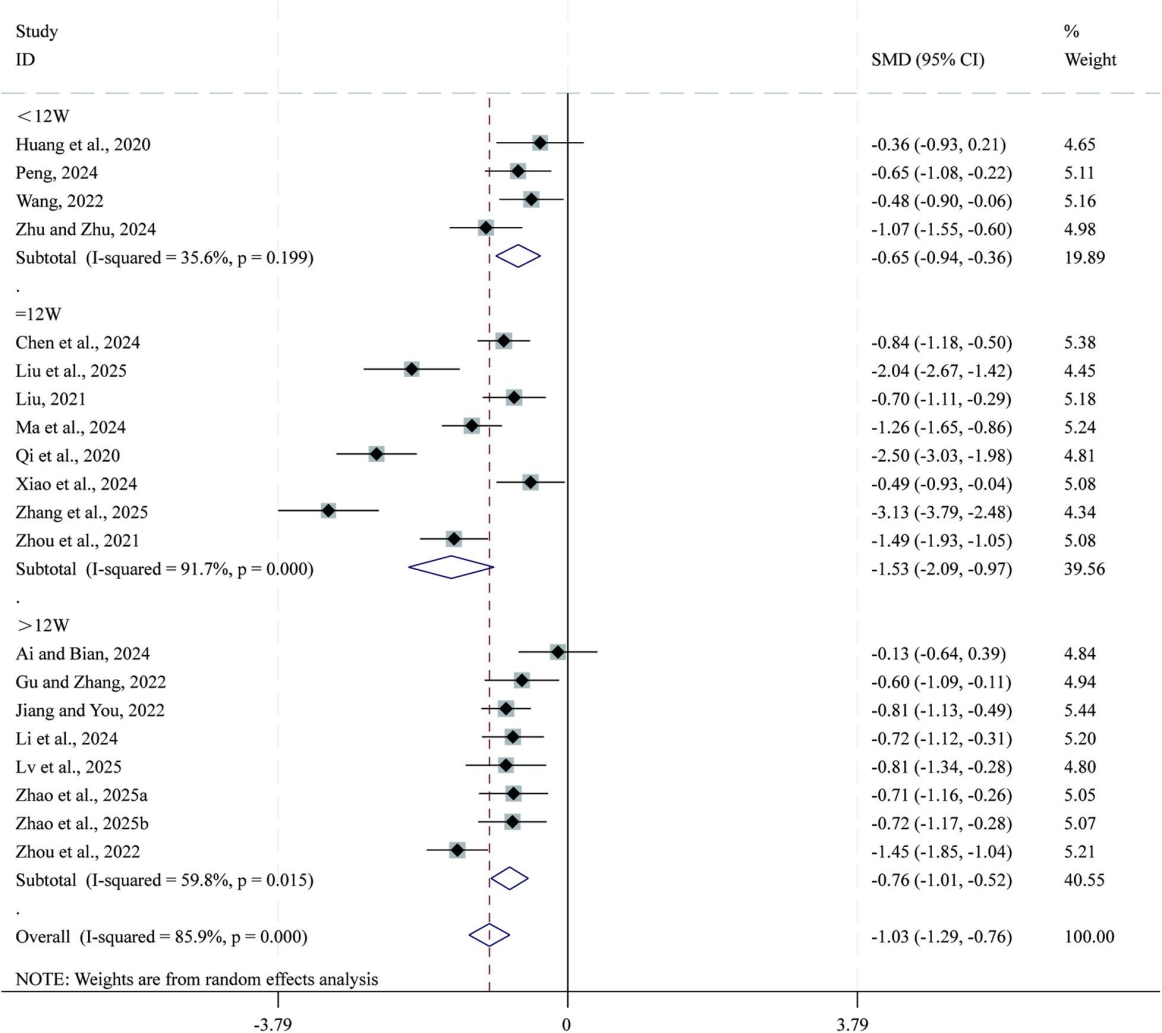
Forest plot for LVEDD.

Total 20 studies (23–25, 28–30, 36–38, 40, 42, 44, 49, 54–56, 59, 63, 66, 68) reported data on LVEDD. Given the substantial heterogeneity across studies (*I²* = 85.9%, *P* = 0.000), a random-effects model was applied for the meta-analysis. Compared with CPT, SBE significantly reduced LVEDD (SMD = −1.03, 95% CI: −1.29 to −0.76, *P* = 0.000; Figure 4). Subgroup analysis based on intervention duration indicated that the greatest reduction in LVEDD occurred in the 12-week intervention subgroup (SMD = −1.53, 95% CI: −2.09 to −0.97, *P* = 0.000), followed by the >12-week subgroup (SMD = −0.76, 95% CI: −1.01 to −0.52, *P* = 0.000), whereas the <12-week subgroup showed comparatively smaller reductions (SMD = −0.65, 95% CI: −0.94 to −0.36, *P* = 0.000).

#### 3.3.3 LVESD

**Figure 5:**
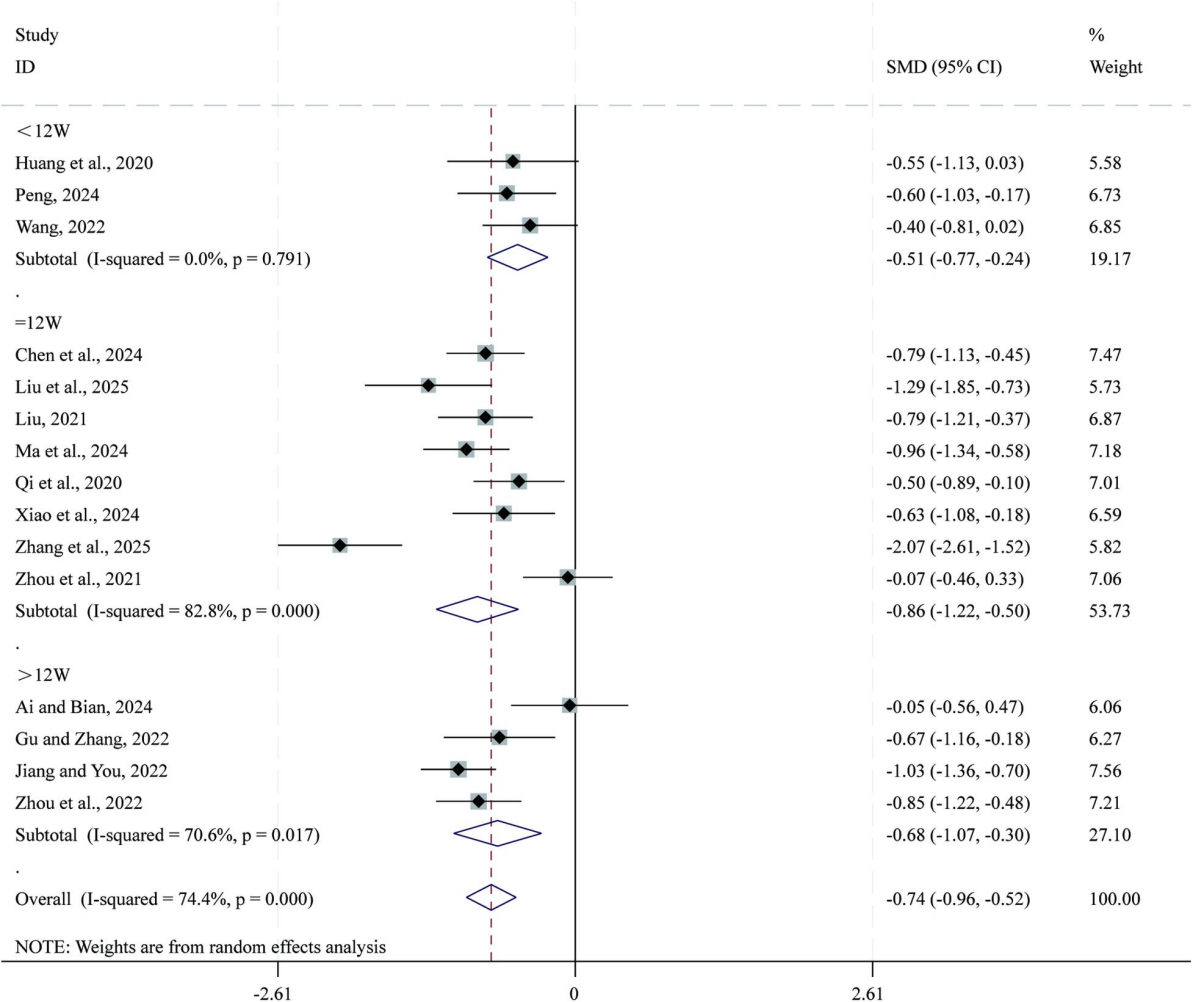
Forest plot for LVESD.

Total 15 studies (25, 29, 36–38, 42, 44, 49, 54–56, 59, 63, 66, 68) reported data on LVESD. Given the substantial heterogeneity across studies (*I²* = 74.4%, *P* = 0.000), a random-effects model was applied for the meta-analysis. Compared with CPT, SBE significantly reduced LVESD (SMD = −0.74, 95% CI: −0.96 to −0.52, *P* = 0.000; Figure 5). Subgroup analysis based on intervention duration indicated that the greatest reduction in LVESD occurred in the 12-week intervention subgroup (SMD = −0.86, 95% CI: −1.22 to −0.50, *P* = 0.000), followed by the >12-week subgroup (SMD = −0.68, 95% CI: −1.07 to −0.30, *P* = 0.001), whereas the <12-week subgroup showed comparatively smaller reductions (SMD = −0.51, 95% CI: −0.77 to −0.24, *P* = 0.000).

#### 3.3.4 6-MWD

**Figure 6:**
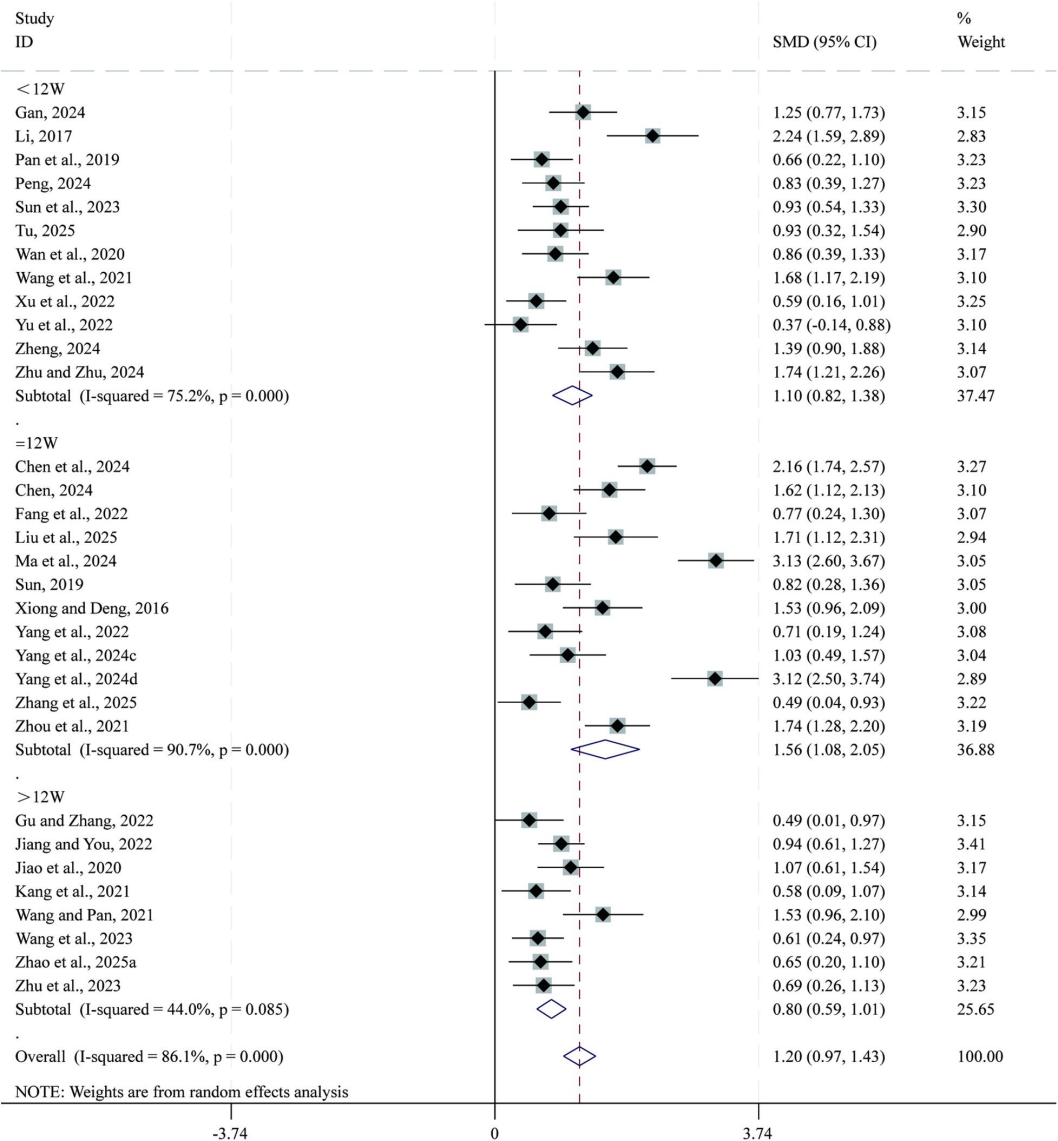
Forest plot for 6-MWD.

Total 32 studies (24, 25, 27, 29–33, 37, 38, 41–43, 45–47, 50, 51, 53, 55–57, 59, 61, 62, 64, 65, 67, 69–72) reported data on 6-MWD. Given the substantial heterogeneity across studies (*I²* = 86.1%, *P* = 0.000), a random-effects model was applied for the meta-analysis. Compared with CPT, SBE significantly improved 6-MWD (SMD = 1.20, 95% CI: 0.97–1.43, *P* = 0.000; Figure 6). Subgroup analysis based on intervention duration indicated that the greatest improvement in 6-MWD occurred in the 12-week intervention subgroup (SMD = 1.56, 95% CI: 1.08–2.05, *P* = 0.000), followed by the <12-week subgroup (SMD = 1.106, 95% CI: 0.82–1.38, *P* = 0.000), whereas the >12-week subgroup showed comparatively smaller improvements (SMD = 0.80, 95% CI: 0.59–1.01, *P* = 0.000).

#### 3.3.5 BNP

**Figure 7:**
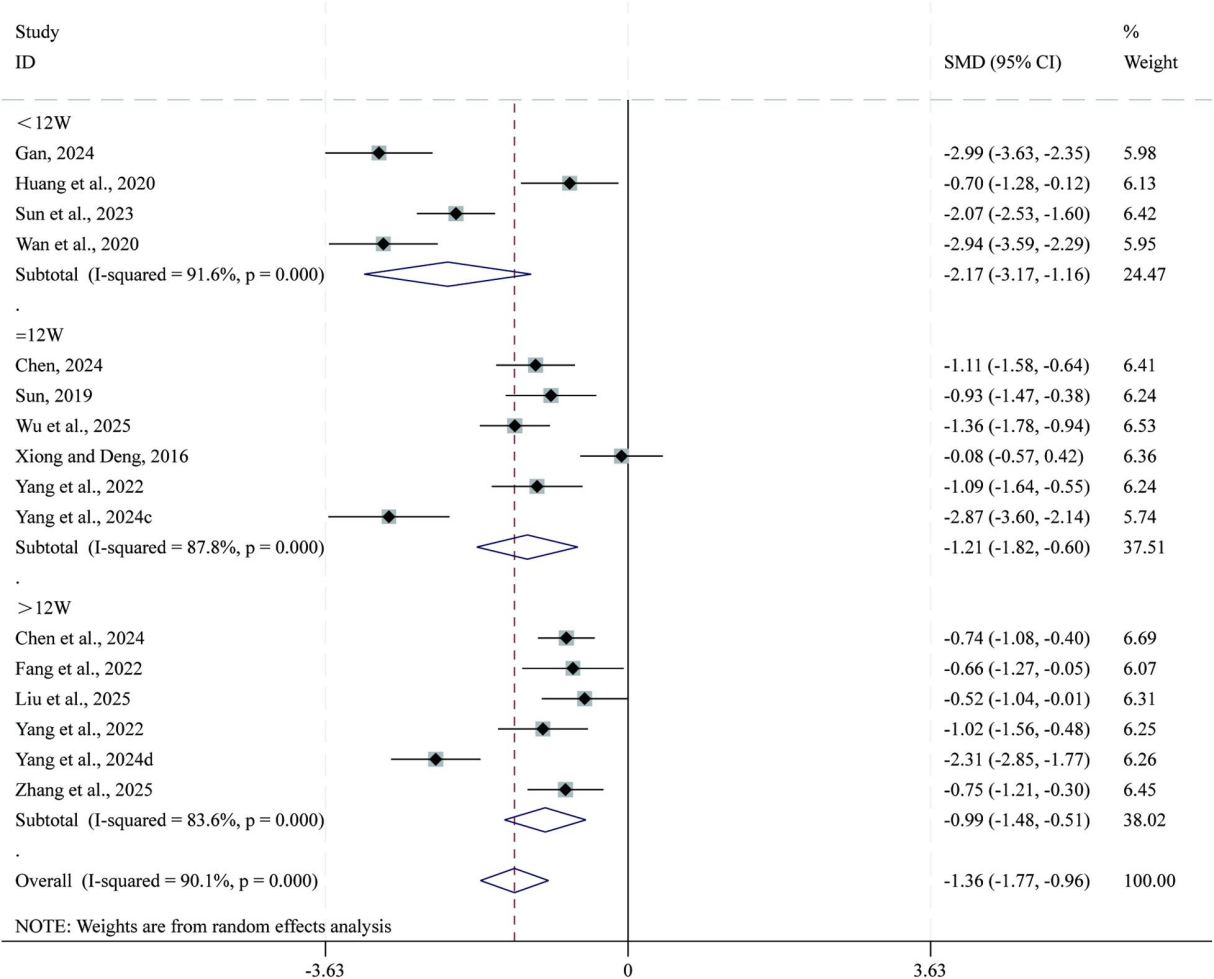
Forest plot for BNP.

Total 11 studies (25, 29, 32, 41, 42, 44, 47, 51, 57, 65, 68) reported data on BNP. Given the substantial heterogeneity across studies (*I²* = 90.1%, *P* = 0.000), a random-effects model was applied for the meta-analysis. Compared with CPT, SBE significantly reduced BNP (SMD = −1.36, 95% CI: −1.77 to −0.96, *P* = 0.000; Figure 7). Subgroup analysis based on intervention duration indicated that the greatest reduction in BNP occurred in the <12-week intervention subgroup (SMD = −2.17, 95% CI: −3.17 to −1.16, *P* = 0.000), followed by the 12-week subgroup (SMD = −1.21, 95% CI: −1.82 to −0.60, *P* = 0.000), whereas the >12-week subgroup showed comparatively smaller reductions (SMD = −0.99, 95% CI: −1.48 to −0.51, *P* = 0.000).

#### 3.3.6 NT-pro BNP

**Figure 8:**
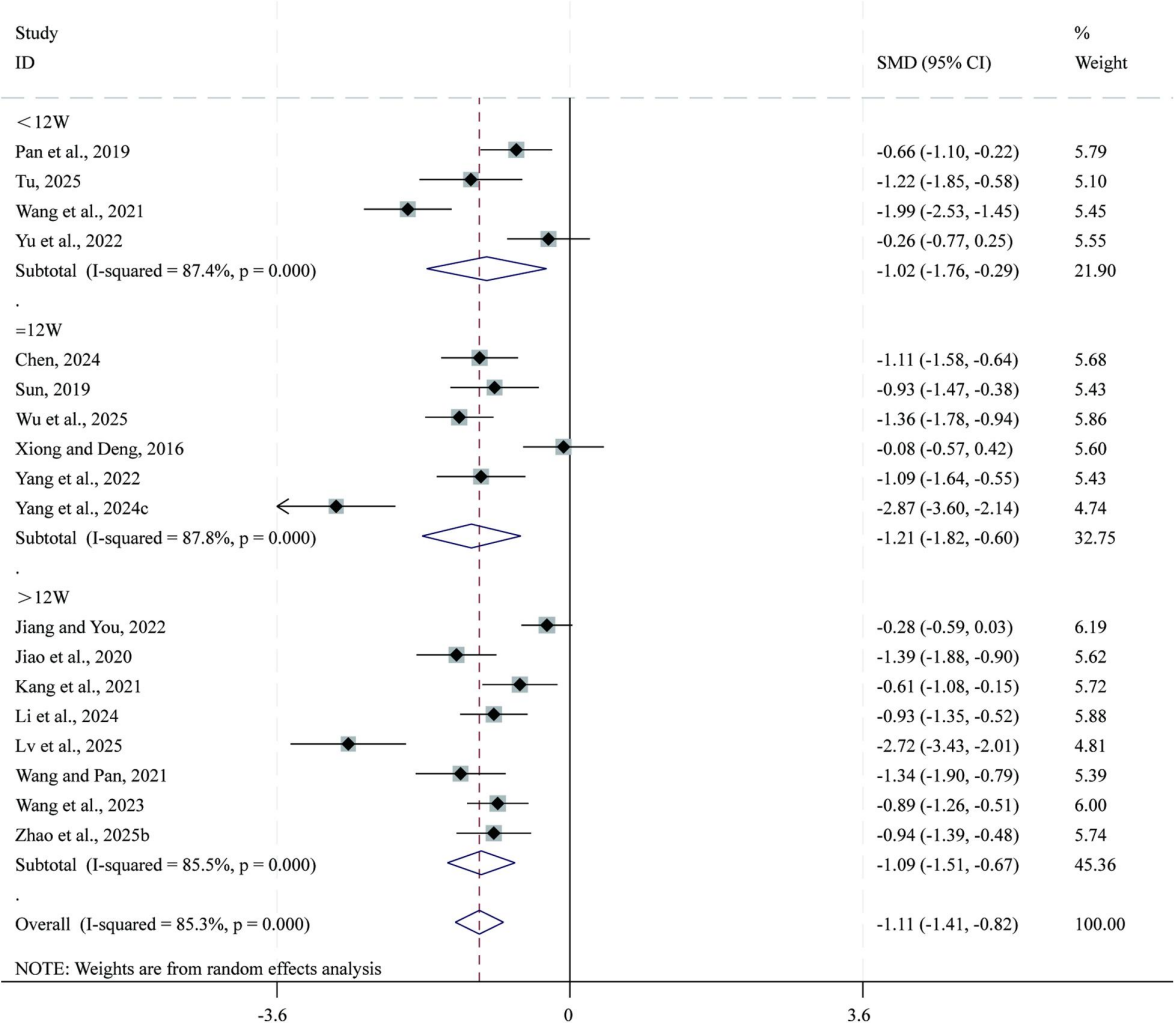
Forest plot for NT-pro BNP.

Total 18 studies (23, 26–28, 33, 40, 43, 46, 50, 51, 55, 61, 62, 64, 67, 69, 70, 72) reported data on NT-pro BNP Given the substantial heterogeneity across studies (*I*^2^ = 85.3%, *P* = 0.000), a random-effects model was applied for the meta-analysis. Compared with CPT, SBE significantly reduced NT-pro BNP (SMD = −1.11, 95% CI: −1.41 to −0.82, *P* = 0.000; Figure 8). Subgroup analysis based on intervention duration indicated that the greatest reduction in NT-pro BNP occurred in the 12-week intervention subgroup (SMD = −1.21, 95% CI: −1.82 to −0.60, *P* = 0.000), followed by the >12-week subgroup (SMD = −1.09, 95% CI: −1.51 to −0.67, *P* = 0.000), whereas the <12-week subgroup showed comparatively smaller reductions (SMD = −1.02, 95% CI: −1.76 to −0.29, *P* = 0.006).

#### 3.3.7 MLHFQ total score

**Figure 9:**
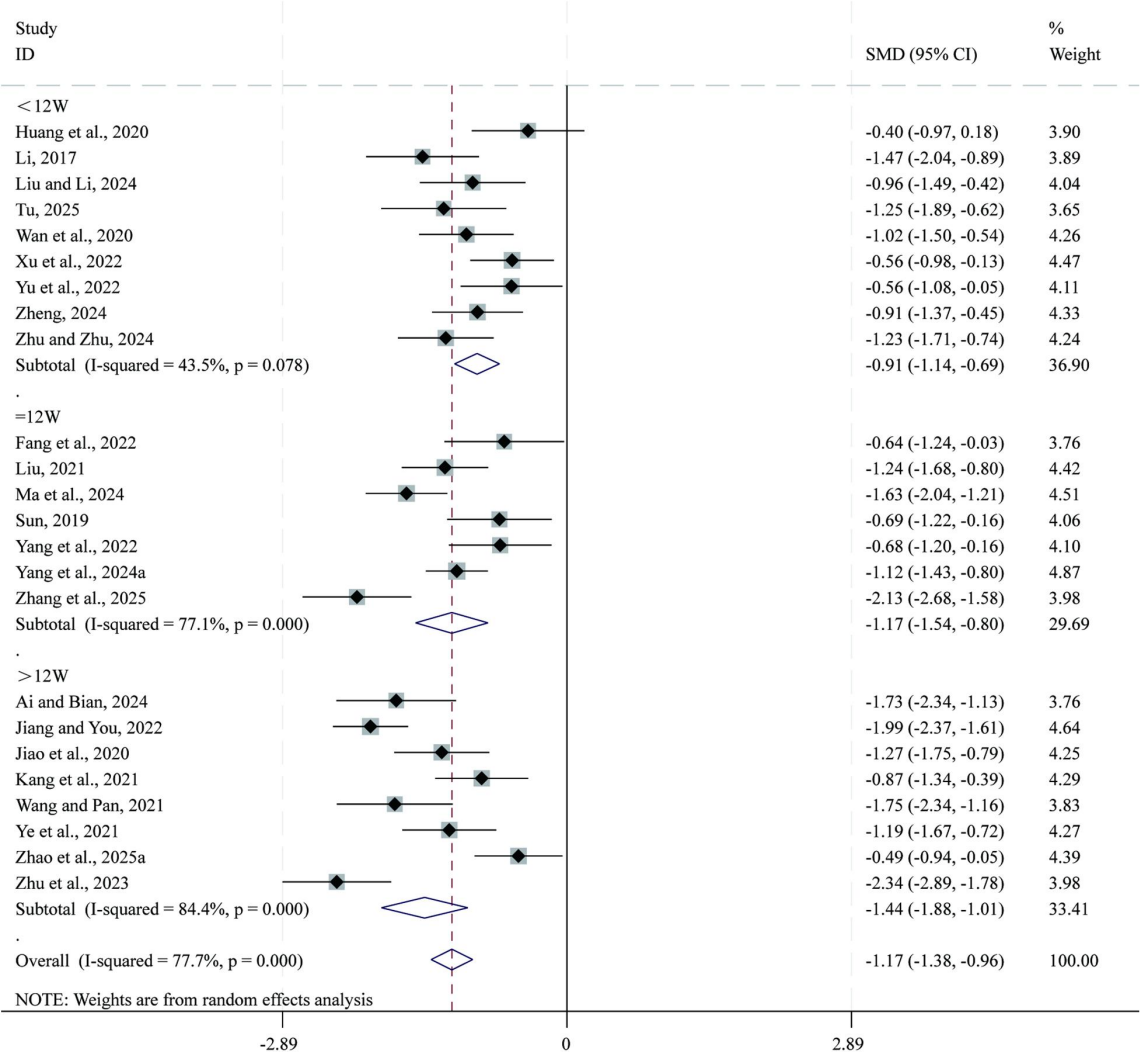
Forest plot for MLHFQ total score.

Total 24 studies (24, 25, 27, 30, 31, 35, 38, 39, 44, 45, 50, 51, 53, 55, 57, 60, 61, 63–65, 67–69, 71) reported data on MLHFQ total score. Given the substantial heterogeneity across studies (*I²* = 77.7%, *P* = 0.000), a random-effects model was applied for the meta-analysis. Compared with CPT, SBE significantly reduced MLHFQ total score (SMD = −1.17, 95% CI: −1.38 to −0.96, *P* = 0.000; Figure 9). Subgroup analysis based on intervention duration indicated that the greatest reduction in MLHFQ total score occurred in the >12-week intervention subgroup (SMD = −1.44, 95% CI: −1.88 to −1.01, *P* = 0.000), followed by the 12-week subgroup (SMD = −1.17, 95% CI: −1.54 to −0.80, *P* = 0.000), whereas the <12-week subgroup showed comparatively smaller reductions (SMD = −0.91, 95% CI: −1.14 to −0.69, *P* = 0.000).

#### 3.3.8 Physical dimensions

**Figure 10:**
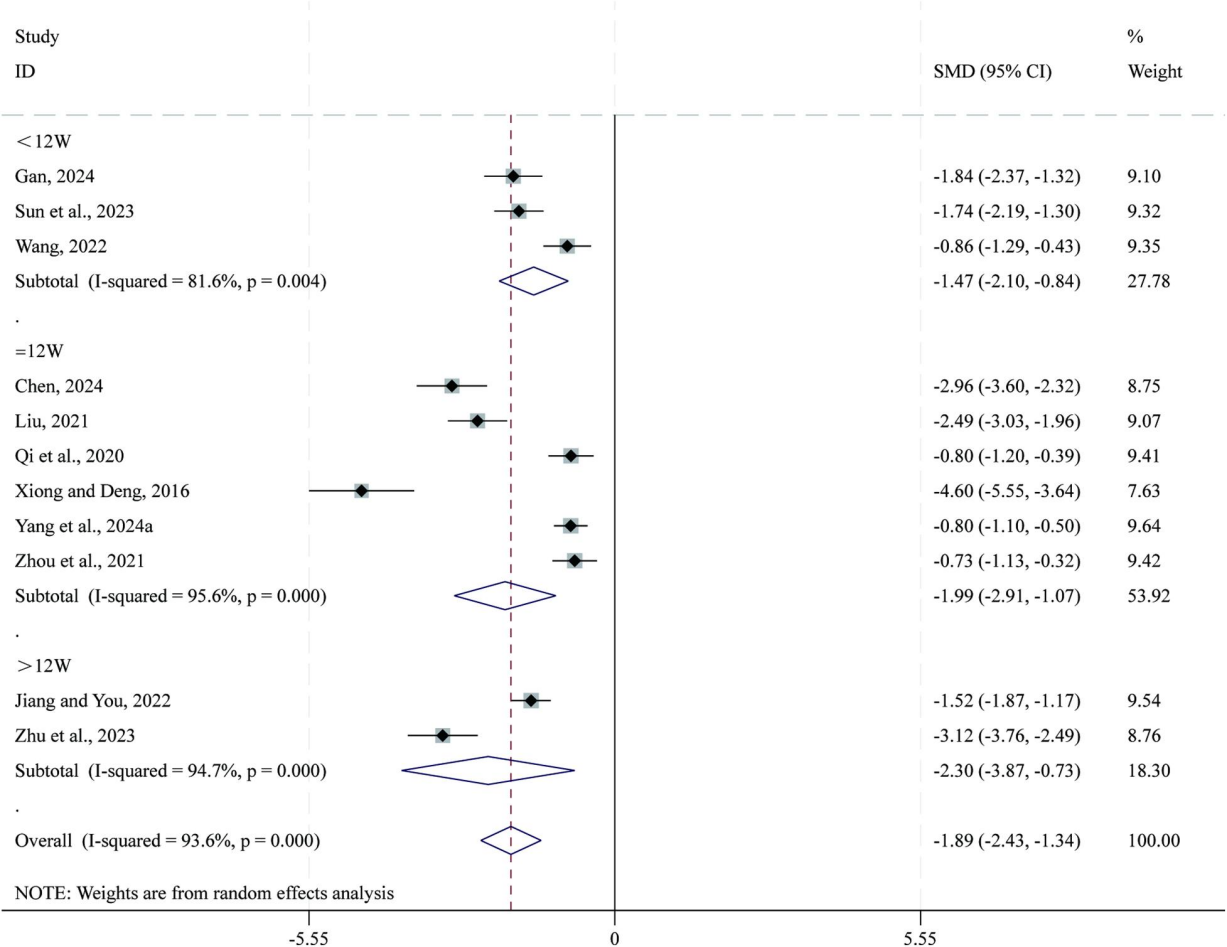
Forest plot for physical dimensions.

Total 11 studies (35, 41, 43, 45, 47, 54, 55, 59, 63, 66, 72) reported data on physical dimensions. Given the substantial heterogeneity across studies (*I*^2^ = 93.6%, *P* = 0.000), a random-effects model was applied for the meta-analysis. Compared with CPT, SBE significantly reduced physical dimensions (SMD = −1.89, 95% CI: −2.43 to −1.34, *P* = 0.000; Figure 10). Subgroup analysis based on intervention duration indicated that the greatest reduction in physical dimensions occurred in the >12-week intervention subgroup (SMD = −2.30, 95% CI: −3.87 to −0.73, *P* = 0.000), followed by the 12-week subgroup (SMD = −1.99, 95% CI: −2.91 to −1.07, *P* = 0.000), whereas the <12-week subgroup showed comparatively smaller reductions (SMD = −1.47, 95% CI: −2.10 to −0.84, *P* = 0.004).

#### 3.3.9 Psychological dimensions

**Figure 11:**
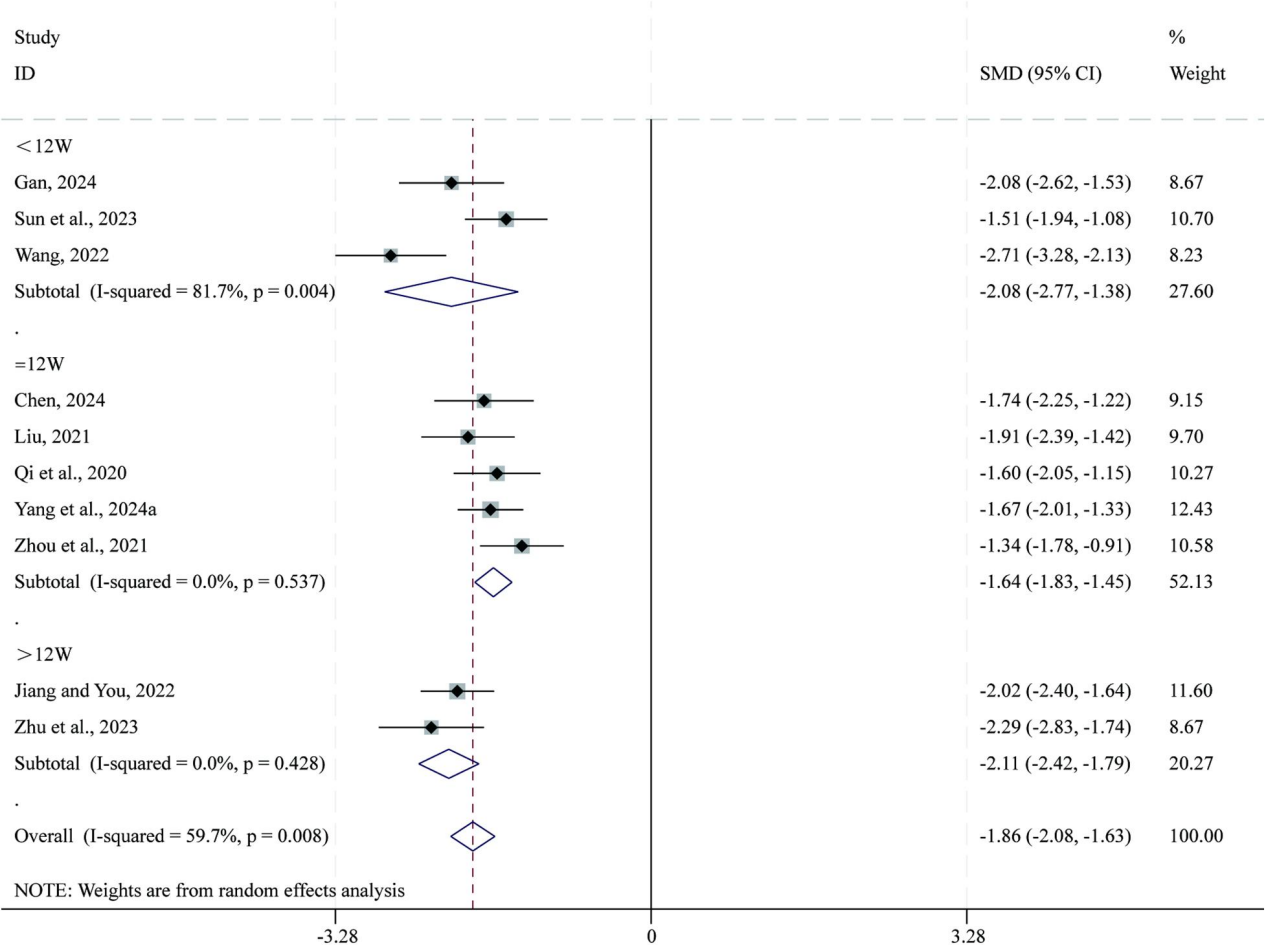
Forest plot for psychological dimensions.

Total 10 studies (35, 41, 43, 45, 47, 54, 55, 59, 63, 66) reported data on psychological dimensions. Given the substantial heterogeneity across studies (*I*^2^ = 59.7%, *P* = 0.008), a random-effects model was applied for the meta-analysis. Compared with CPT, SBE significantly reduced psychological dimensions (SMD = −1.86, 95% CI: −2.08 to −1.63, *P* = 0.000; Figure 11). Subgroup analysis based on intervention duration indicated that the greatest reduction in psychological dimensions occurred in the >12-week intervention subgroup (SMD = −2.11, 95% CI: −2.42 to −1.79, *P* = 0.000), followed by the <12-week subgroup (SMD = −2.08, 95% CI: −2.77 to −1.38, *P* = 0.000), whereas the 12-week subgroup showed comparatively smaller reductions (SMD = −1.64, 95% CI: −1.83 to −1.45, *P* = 0.000).

#### 3.3.10 Other dimensions

**Figure 12:**
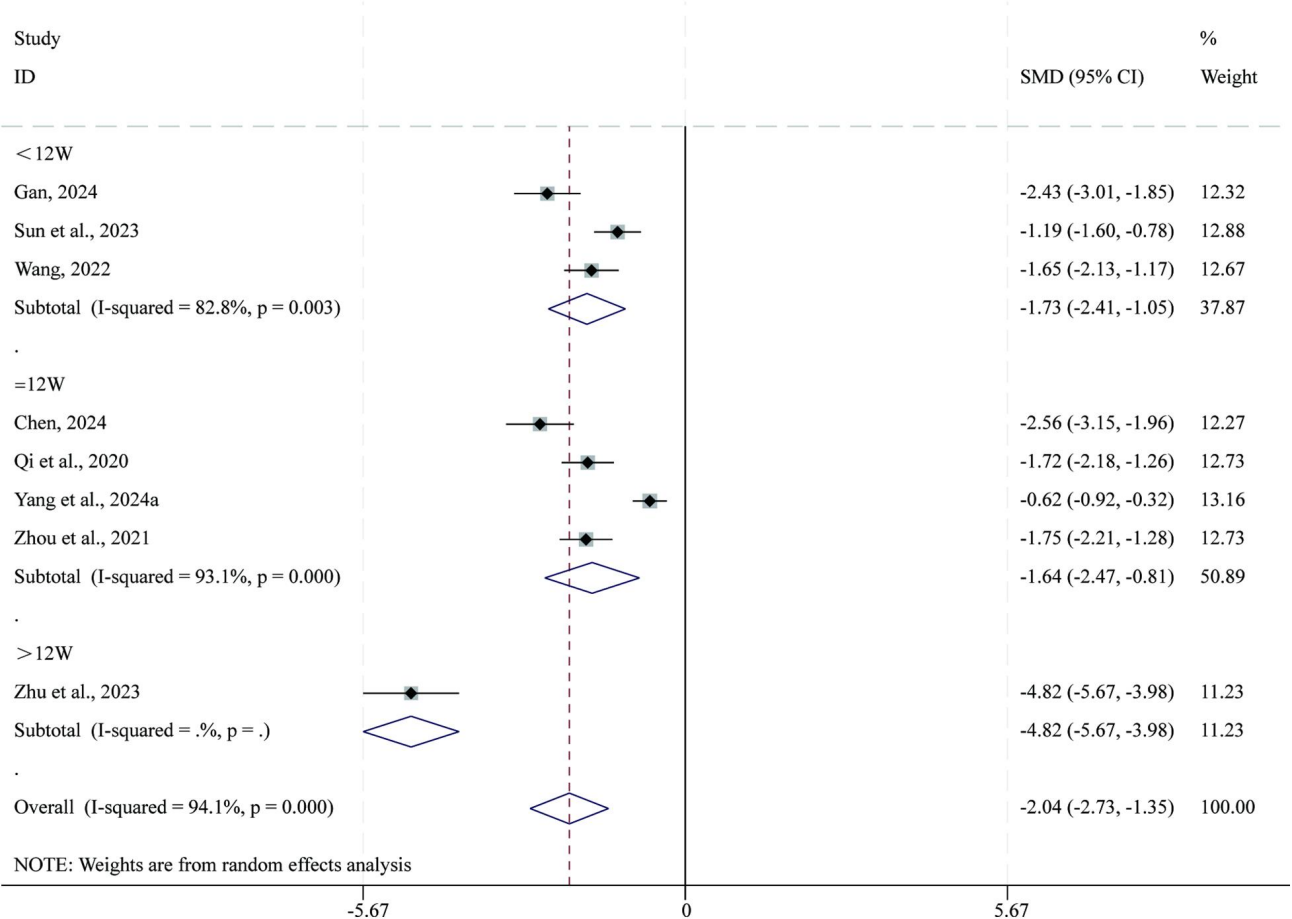
Forest plot for other dimensions.

Total 8 studies (35, 41, 43, 45, 47, 54, 59, 66) reported data on other dimensions. Given the substantial heterogeneity across studies (*I*^2^ = 94.1%, *P* = 0.000), a random-effects model was applied for the meta-analysis. Compared with CPT, SBE significantly reduced other dimensions (SMD = −2.04, 95% CI: −2.73 to −1.35, *P* = 0.000; Figure 12). Subgroup analysis based on intervention duration indicated that the greatest reduction in other dimensions occurred in the >12-week intervention subgroup (SMD = −4.82, 95% CI: −5.67 to −3.98, *P* = 0.000), followed by the <12-week subgroup (SMD = −1.73, 95% CI: −2.41 to −1.05, *P* = 0.000), whereas the 12-week subgroup showed comparatively smaller reductions (SMD = −1.64, 95% CI: −2.47 to −0.81, *P* = 0.000).

#### 3.3.11 Clinical efficacy

**Figure 13:**
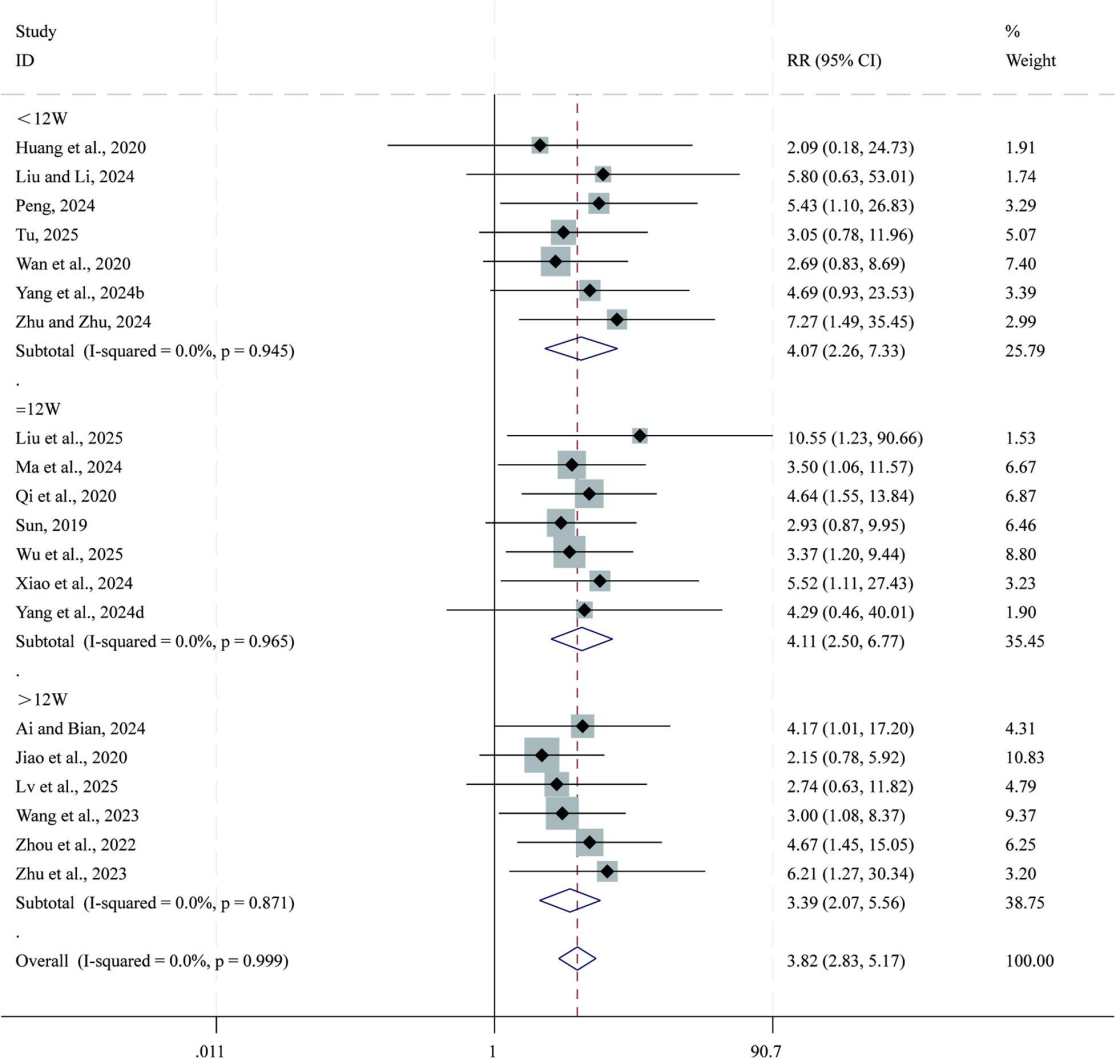
Forest plot for clinical efficacy.

Total 20 studies (26–30, 32, 34, 36–39, 44–46, 49, 65–69) reported data on clinical efficacy. Given the low heterogeneity across studies (*I²* = 0.0%, *P* = 0.999), a fixed-effects model was applied for the meta-analysis. Compared with CPT, SBE significantly improved clinical efficacy (RR = 3.82, 95% CI: 2.83–5.17, *P* = 0.000; Figure 13). Subgroup analysis based on intervention duration indicated that the greatest improvement in clinical efficacy occurred in the 12-week intervention subgroup (RR = 4.11, 95% CI: 2.50–6.77, *P* = 0.000), followed by the <12-week subgroup (RR = 4.07, 95% CI: 2.26–7.33, *P* = 0.000), whereas the >12-week subgroup showed comparatively smaller improvements (RR = 3.39, 95% CI: 2.07–5.56, *P* = 0.000).

### 3.4 Sensitivity analysis

**Figure 14:**
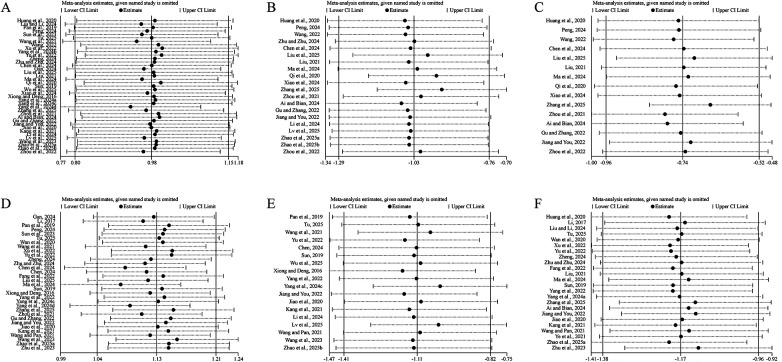
The results of sensitivity analysis. **(A)** LVEF. **(B)** LVEDD. **(C)** LVESD. **(D)** 6-MWD. **(E)** NT-pro BNP. **(F)** MLHFQ total score.

Sensitivity analyses were performed for LVEF (Figure 14A), LVEDD (Figure 14B), LVESD (Figure 14C), 6-MWD (Figure 14D), NT-pro BNP (Figure 14E), and MLHFQ total score (Figure 14F) by sequentially excluding individual studies. The pooled results showed minimal variation in effect size or significance, indicating that the findings were stable and reliable.

### 3.5 Publication bias

**Figure 15:**
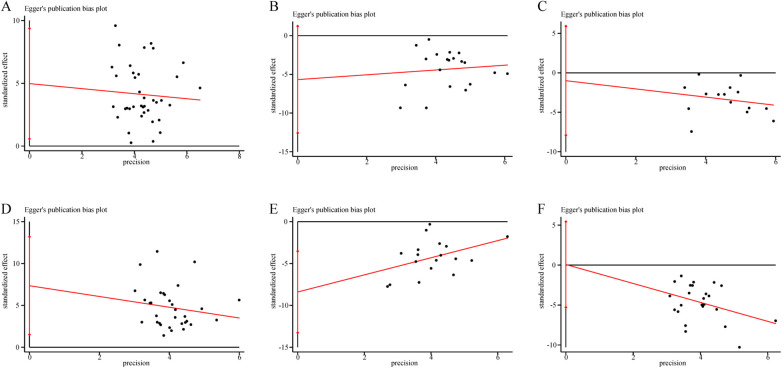
The results of publication bias. **(A)** LVEF. **(B)** LVEDD. **(C)** LVESD. **(D)** 6-MWD. **(E)** NT-pro BNP. **(F)** MLHFQ total score.

Publication bias was assessed for LVEF, LVEDD, LVESD, 6-MWD, NT-pro BNP, and MLHFQ using Egger's test. Egger's test suggested potential publication bias for LVEF (Figure 15A, *P* = 0.028), 6-MWD (Figure 15D, *P* = 0.015), and NT-pro BNP (Figure 15E, *P* = 0.003), while no significant bias was found for LVEDD (Figure 15B, *P* = 0.101), LVESD (Figure 15C, *P* = 0.760), or MLHFQ (Figure 15F, *P* = 0.978). To further verify these results, the trim-and-fill method was applied to the indicators showing bias (Supplementary Material S4). The adjusted pooled effects showed minimal change, indicating that although minor publication bias might exist, the overall findings remained robust and credible.

## 4 Discussion

### 4.1 Summary of findings

The results of this systematic review and meta-analysis demonstrate that SBE, as an adjunctive intervention for patients with CHF, exerts significant benefits in improving both cardiac function and QoL. In terms of cardiac function, SBE markedly increased LVEF and 6-MWD, indicating enhanced myocardial contractility and exercise tolerance. Meanwhile, it significantly reduced LVEDD, LVESD, BNP, and NT-pro BNP levels, suggesting attenuation of ventricular remodeling and improvement in cardiac load. Regarding QoL, SBE significantly decreased total and domain scores (physical, psychological, and other) of the MLHFQ, reflecting not only alleviation of physical symptoms and functional impairment but also mitigation of psychological distress and social limitations. Moreover, when combined with CPT, SBE further enhanced overall clinical efficacy, underscoring its potential as a complementary rehabilitation approach.

Sensitivity analyses revealed that the pooled effect sizes for major outcomes remained stable after sequential exclusion of individual studies, indicating the robustness and reliability of the meta-analytic results. Egger's tests suggested minor publication bias for LVEF, 6-MWD, and NT-pro BNP, whereas no significant bias was observed for LVEDD, LVESD, or MLHFQ, implying an overall low risk of publication bias. To explore the potential influence of intervention duration, subgroup analyses were performed based on treatment length. The results indicated that 12 weeks of SBE produced the greatest improvement in cardiac function, whereas interventions lasting more than 12 weeks yielded the most pronounced benefits in QoL. This pattern may reflect the distinct physiological and psychosocial adaptation processes underlying exercise rehabilitation. Moderate-intensity mind-body SBE typically induce significant autonomic remodeling, anti-inflammatory, and antioxidative adaptations within 8–12 weeks, leading to enhanced myocardial contractility and metabolic efficiency. Beyond this period, although further physiological gains may plateau, continued practice helps consolidate behavioral adherence, emotional regulation, and psychosocial well-being, resulting in sustained improvement in QoL. Overall, SBE represents a safe, cost-effective, and sustainable adjunctive therapy that supports both functional recovery and long-term holistic rehabilitation in patients with CHF.

### 4.2 Cardioprotective mechanisms of SBE

The core pathophysiological mechanisms underlying CHF involve excessive activation of the renin-angiotensin-aldosterone system (RAAS), heightened sympathetic nervous activity, and sustained upregulation of pro-inflammatory cytokines (73). These abnormalities, through neurohormonal imbalance and immune-inflammatory cascades, jointly promote cardiomyocyte hypertrophy, apoptosis, and fibrosis, ultimately resulting in ventricular remodeling and progressive cardiac dysfunction. SBE, characterized by slow, coordinated movements integrated with deep breathing, contributes to autonomic regulation by restoring the balance between the sympathetic and parasympathetic nervous systems (74). It has been shown to reduce circulating norepinephrine levels, enhance vagal tone, and thereby suppress sympathetic overactivation, which in turn decreases cardiac preload and myocardial oxygen consumption (75). Furthermore, SBE exerts potent anti-inflammatory and antioxidative effects, potentially through inhibition of the NF-*κ*B signaling pathway and upregulation of endogenous antioxidant enzymes such as SOD and GSH-Px (18). These effects collectively alleviate myocardial inflammation and fibrotic progression. Improvement in endothelial function represents another crucial cardioprotective mechanism. Evidence suggests that SBE can upregulate NOS expression and increase NO bioavailability, enhancing vasodilation and microcirculatory perfusion (19). Activation of the L-arginine-NO signaling pathway further reduces peripheral vascular resistance and optimizes cardiac loading conditions (76). In addition, the “integration of movement and stillness” and “coordination of body and mind” inherent in SBE can help alleviate anxiety and depression while stabilizing the hypothalamic-pituitary-adrenal (HPA) axis, thereby promoting cardiac recovery from both psychological and physiological dimensions (77, 78). Collectively, SBE provides both evidence-based support and modern physiological rationale for its role as an integrative rehabilitation approach in CHF.

### 4.3 Comparison with previous studies

Previous meta-analyses by Mei et al. (79) and Yang et al. (80) investigated the effects of Baduanjin on CHF, the present study provides a more systematic and methodologically refined evaluation. First, this study is the first to distinguish between standing and sitting forms of Baduanjin, including only trials that adopted SBE. This distinction minimized the potential confounding effects caused by variations in exercise intensity, posture, and movement patterns. The results demonstrated that SBE produced more pronounced improvements in both cardiac function and QoL than those reported in earlier mixed-form analyses. Second, through subgroup analyses, this study identified a time-dependent relationship between intervention duration and therapeutic efficacy, showing that a 12-week intervention produced the greatest improvement in cardiac function, while interventions longer than 12 weeks resulted in the most pronounced enhancement in QoL. This finding suggests that the rehabilitative benefits of SBE may depend on the physiological adaptation period required for neurocardiac and behavioral conditioning. Finally, unlike previous studies that primarily focused on cardiac function outcomes, the current meta-analysis incorporated QoL (MLHFQ total and subdomain scores) as co-primary endpoints. This comprehensive approach provided a more holistic understanding of SBE's multidimensional rehabilitation value—encompassing physiological, psychological, and social domains. Overall, this study collectively reinforces the clinical potential of SBE as a safe, feasible, and multidimensionally beneficial exercise-based rehabilitation strategy for patients with CHF.

### 4.4 Strengths and limitations

Impaired cardiac function and reduced quality of life represent two major challenges in the long-term management of CHF. Cardiac pump dysfunction leads to diminished exercise capacity and excessive neurohormonal activation, while sustained declines in QoL are closely associated with depression, anxiety, and increased risk of rehospitalization (81–83). In recent years, exercise-based rehabilitation has been shown to improve cardiac function, exercise tolerance, and overall well-being in patients with CHF (84); however, the optimal modality and duration of intervention remain uncertain. This study comprehensively evaluated the effects of SBE on cardiac function and QoL in patients with CHF, with several notable strengths. First, it is the first meta-analysis to distinguish standing from sitting Baduanjin, including only standing Baduanjin RCTs to minimize confounding from differences in exercise intensity. Second, a subgroup analysis based on intervention duration (<12 weeks, =12 weeks, >12 weeks) revealed the time-dependent characteristics of its cardiorehabilitative effects. Third, by integrating both cardiac function and QoL as co-primary outcomes, the study established a more comprehensive assessment framework, providing systematic evidence for the clinical application of SBE in CHF rehabilitation.

However, several limitations should be acknowledged. First, all included studies were conducted in China, with relatively homogeneous populations, which may limit the generalizability of the findings to other regions and ethnic groups. Second, some trials lacked methodological rigor, particularly regarding allocation concealment and blinding, which may introduce selection or detection bias. Third, the included studies did not distinguish between heart failure subtypes—such as reduced ejection fraction (HFrEF), mildly reduced ejection fraction (HFmrEF), and preserved ejection fraction (HFpEF)—nor did they consistently stratify outcomes according to NYHA functional class II or III, thereby limiting the ability to assess differential effects across CHF phenotypes and disease severity. In addition, the etiology of CHF (e.g., ischemic, hypertensive, or other causes) was not uniformly reported, precluding etiology-specific analyses. Fourth, inconsistencies existed in training frequency, session duration, instructional formats, and supervision quality of SBE, and most studies did not include objective measures of exercise intensity (e.g., heart rate zones or Borg scale), which may affect reproducibility and dose-response analysis. Fifth, standard pharmacological regimens varied across studies, and such heterogeneity could have influenced the observed outcomes. Finally, most trials had short follow-up periods and lacked data on long-term outcomes such as rehospitalization rates, mortality, and adverse events, preventing comprehensive evaluation of long-term safety and sustainability. Future research should therefore focus on multicenter, large-sample, rigorously designed prospective studies with extended follow-up periods. Further refinement of heart failure subtype stratification, etiological classification, and standardized exercise prescription parameters is warranted to clarify the cardioprotective mechanisms and clinical value of SBE in the integrated management of CHF.

### 4.5 Implications

In light of the aforementioned limitations, future research should advance the study of SBE. First, it is essential to establish a unified standard framework for Baduanjin-based cardiac rehabilitation. This includes defining the training frequency, session duration, postural specifications, and exercise intensity monitoring indicators (e.g., heart rate zones, Borg Rating of Perceived Exertion), thereby creating a quantifiable and reproducible exercise prescription model. Second, future studies should adopt a stratified design based on heart failure subtypes—HFrEF, HFmrEF, and HFpEF—to explore the differential efficacy, safety boundaries, and adaptability of SBE across distinct pathophysiological categories, ultimately facilitating precision rehabilitation. Third, there is a pressing need for multicenter, large-sample, and long-term RCTs conducted in diverse populations and geographic regions. Such studies should verify the sustained effects of SBE on cardiac function, rehospitalization rate, and mortality, while systematically documenting adverse events to evaluate long-term safety and clinical sustainability. Fourth, mechanistic investigations should be strengthened by integrating heart rate variability (HRV) analysis, inflammatory cytokine profiling, vascular endothelial function assessment, and multi-omics approaches. These studies can help clarify the biological underpinnings of SBE in regulating autonomic balance, anti-inflammatory and antioxidative pathways, and myocardial remodeling. Moreover, objective multimodal assessments using cardiac magnetic resonance imaging (MRI), cardiopulmonary exercise testing (CPET), and wearable monitoring devices should be incorporated to bridge the gap between clinical efficacy observation and mechanistic validation. In the future, efforts should focus on integrating SBE into modern cardiac rehabilitation systems, promoting it as a scalable, quantifiable, and sustainable model for global heart failure rehabilitation.

## 5 Conclusion

Current evidence suggests that SBE can effectively improve cardiac function in patients with CHF, as reflected by significant increases in LVEF and 6-MWD, and reductions in BNP, NT-pro BNP, and MLHFQ scores. However, given the methodological limitations, heterogeneity of intervention protocols, and geographic concentration of existing studies, future high-quality, multicenter, and long-term RCTs are warranted to further validate these conclusions and consolidate the role of SBE in the comprehensive management of CHF.

## Data Availability

The original contributions presented in the study are included in the article/Supplementary Material, further inquiries can be directed to the corresponding authors.
